# Role of HMGB1/TLR4 and IL-1β/IL-1R1 Signaling Pathways in Epilepsy

**DOI:** 10.3389/fneur.2022.904225

**Published:** 2022-06-28

**Authors:** Shaohui Zhang, Feng Chen, Feng Zhai, Shuli Liang

**Affiliations:** ^1^Functional Neurosurgery Department, National Children's Health Center of China, Beijing Children's Hospital, Capital Medical University, Beijing, China; ^2^Neurosurgery Department, People's Liberation of Army (PLA) General Hospital, Beijing, China; ^3^Beijing Key Laboratory of Major Diseases in Children, Ministry of Education, Functional Neurosurgery Department, National Children's Health Center of China, Beijing Children's Hospital, Capital Medical University, Beijing, China

**Keywords:** high mobility group box1, toll-like receptor, interleukin-1β, interleukin-1receptor1, high mobility group box1, epilepsy

## Abstract

Epilepsy is a chronic disorder of the nervous system characterized by recurrent seizures. Inflammation is one of the six major causes of epilepsy, and its role in the pathogenesis of epilepsy is gaining increasing attention. Two signaling pathways, the high mobility group box-1 (HMGB1)/toll-like receptor 4 (TLR4) and interleukin-1β (IL-1β)/interleukin-1 receptor 1 (IL-1R1) pathways, have become the focus of research in recent years. These two signaling pathways have potential as biomarkers in the prediction, prognosis, and targeted therapy of epilepsy. This review focuses on the association between epilepsy and the neuroinflammatory responses mediated by these two signaling pathways. We hope to contribute further in-depth studies on the role of HMGB1/TLR4 and IL-1β/IL-1R1 signaling in epileptogenesis and provide insights into the development of specific agents targeting these two pathways.

## Introduction

Epilepsy is a common chronic neurological disease, with a worldwide annual incidence and a prevalence rate of 50 in 100,000 and 700 in 100,000, respectively (1). Approximately 70 million people suffer from epilepsy (1, 2). Seizures seriously affect a patient's physical and mental health and create an economic burden on his family and society as a whole. Although anti-seizure medications have been widely used, approximately one-third of patients suffer from drug-refractory epilepsy. Surgery can be performed, but some patients still have poor postoperative outcomes (3). Based on the current situation, it is necessary to explore new mechanisms of epileptogenesis to develop novel therapeutic targets.

The mechanisms of epilepsy are complex, and the neuroinflammatory response is closely related to epilepsy. Inflammatory factors can affect the electrical activity of neurons and glial cells, in addition to causing local inflammatory reactions, while epileptic seizures also cause neuro-inflammatory reactions, further aggravating nerve cell damage, thus forming a vicious cycle involving immune inflammation, epileptic seizures, and brain injury (4, 5). Among the signaling pathways involved in inflammation, high mobility group protein B1 (HMGB1)/toll-like receptor 4 (TLR4) and interleukin-1β (IL-1 β)/interleukin 1 receptor (IL-1R) have received much attention. Here, HMGB1/TLR4 and IL-1β/IL-1R inflammasomes in the pathogenesis of epilepsy are reviewed and future perspectives are outlined.

## Structure and Function of HMGB1/TLR4

**Figure 1:**
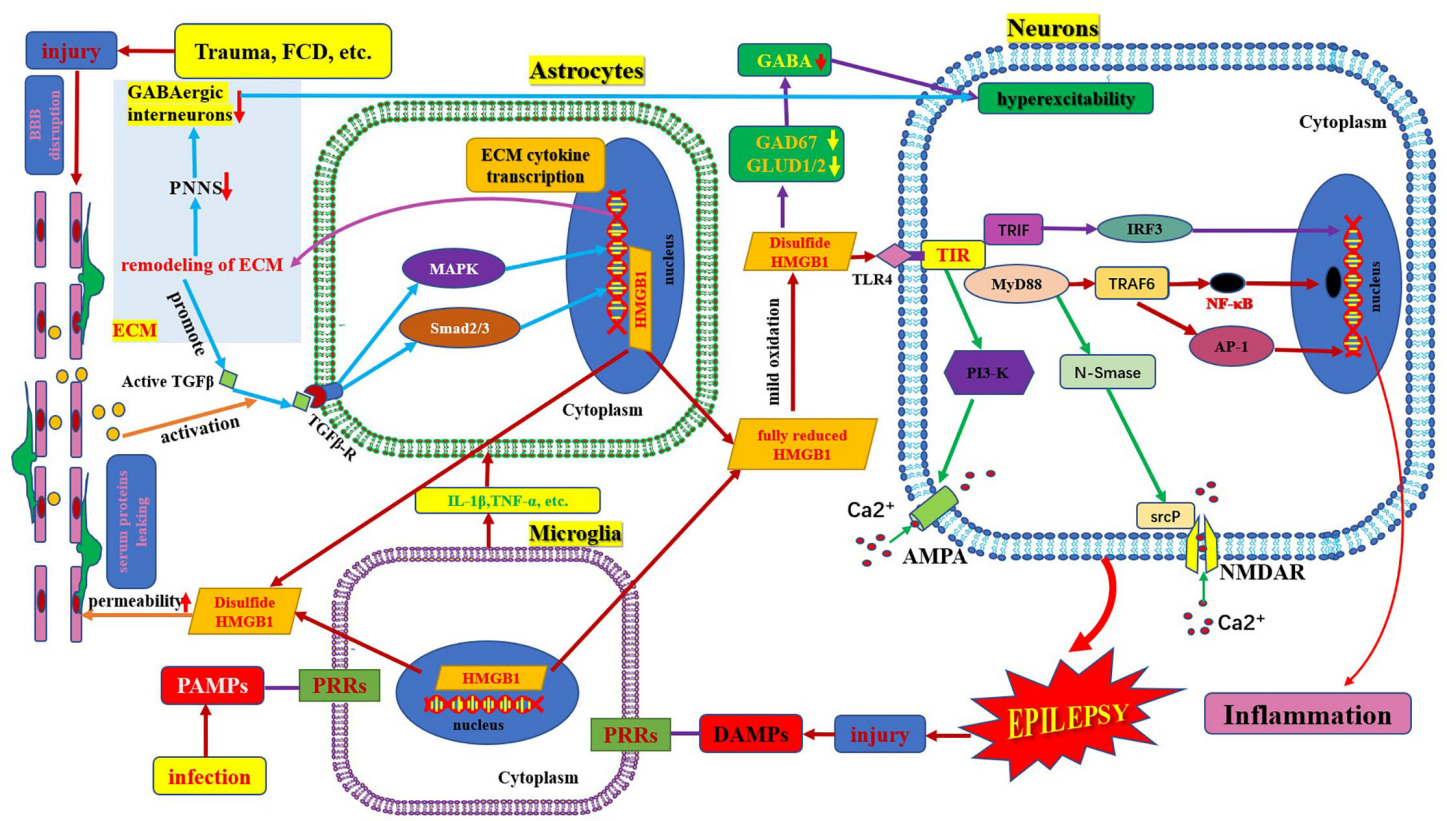
Schematic of HMGB1/TLR-4 pathway and epilepsy. This figure shows the activation and secretion of HMGB1 and its epileptogenic mechanism, in combination with TLR4. AMPA, aminohydroxymethyloxazole propionic acid; BBB, blood–brain barrier; DAMP, danger-associated molecular patterns; FCD, focal cortical dysplasia; GAD 67, glutamate decarboxylase 67; GABA, γ- aminobutyric acid; GLUD, glutamate dehydrogenase; HMGB1, high mobility group box1; IL-1β, interleukin-1β; MyD88, myeloid differentiation factor 88; NMDAR, N-methyl-D-aspartic acid receptor; NF-κB, nuclear factors κB; PAMP, pathogen-associated molecular patterns; PRR, pattern recognition receptors; PI3K, phosphatidylinositol trihydroxykinase; TLR, toll-like receptor; TNF, tumor necrosis factor.

HMGB1 is a non-histone protein located in the nucleus, which binds to DNA and is involved in DNA transcription, translation, and repair (6). HMGB1 is a chain-like structural protein made up of 215 amino acids, containing a sour tail (C-terminus) and two DNA-binding domains (box A and box B) located in the L configuration (N-terminus) (7). Box B, as a proinflammatory site, can be recognized by TLRs and the receptor for advanced glycation end products inducing HMGB1 to exhibit proinflammatory activity. Box A is an anti-inflammatory response site that can competitively inhibit the proinflammatory activity of HMGB1; however, both box B and box A can bind to DNA and play a role in folding and distorting the double-stranded DNA (8, 9). The two DNA binding boxes of HMGB1 contain three cysteines (cys23, cys45, and cys106). All three cysteines reside in a fully reduced state with thiol groups (all-thiol HMGB1) in normal cells. It is reported that HMGB1 exists in three isomers: fully reduced HMGB1, disulfide HMGB1, and sulfonyl HMGB1 (8). Fully reduced HMGB1 mainly exists in the nucleus in the form of non-acetylated thiol-HMGB1, which possesses cell migration-inducing activity but lacks cytokine-inducing activity. Fully reduced HMGB1 can be released into the extracellular space from damaged neurons and glial cells. In the extracellular space, non-acetylated thiol HMGB1 is partially oxidized to generate disulfide HMGB1 with a disulfide bond between Cys23 and Cys45; however, Cys106 remains in the reduced form. It is a TLR4 ligand that possesses cytokine-inducing activity but lacks cell migration-induced activity (9). Disulfide HMGB1 binds to TLR4 to initiate a neuroinflammatory response (Figure 1) (10). Complete oxidation of HMGB1 will produce sulfonyl HMGB1, lacking both cell migration-induced activity and cytokine-inducing activity. However, a new finding in tumor biology reveals that sulfonyl HMGB1 exerted anti-inflammatory effects *via* the RAGE signaling pathway, which can recruit immune-competent cells and inhibit cytotoxic cells (11). HMGB1 can be released into the extracellular space from neurons and glial cells in an active or passive manner. In the extracellular space, HMGB1 is mildly oxidized to generate disulfide HMGB1 with a disulfide bond between Cys23 and Cys45; however, Cys106 remains in the reduced form (9). Disulfide HMGB1 binds to TLR4 to initiate a neuroinflammatory response (Figure 1) (9, 10).

TLRs were first identified in 1988 in Drosophila melanogaster, followed by recognition of its homolog, TLR4, in humans in 1997. To date, 13 different types of TLRs (TLR1-TLR13) have been discovered in mammals, among which 10 are present in humans (TLR1-10), including both intracellular and extracellular members (12). They are members of the pattern recognition receptor family that bind to pathogen-associated molecular patterns to initiate innate immune responses. TLR4 is a receptor of innate immunity and a lipopolysaccharide (LPS) sensor that is widely expressed in several immune and non-immune cells of the mammalian host. Of note, both non-transformed cells (endothelial and epithelial cells, fibroblasts, glial cells, neurons, and neural progenitor cells) and transformed cells (neoplastic) in the body have been detected to express TLRs (8). It is widely expressed in the central nervous system, including microglia, astrocytes, and neurons (13). TLR4 contains intracellular, transmembrane, and extracellular domains. The intracellular transmembrane domain, also known as the Toll-IL-1R (TIR) domain, plays a role in signal transduction, and the extracellular domain consists of leucine-rich repeats (LRRs) (14). The main functions of TLR4 include the regulation of cytokine secretion and microglial phagocytic activity. TLR4 signaling in the brain drives autoimmune responses and initiates neuroinflammation, which plays an important role in a variety of brain disorders (e.g., cerebrovascular disease, brain tumors, and epilepsy) (15). HMGB1 is mainly involved in the pathophysiology of epilepsy by interacting with the primary receptor TLR4.

## Mechanisms Underlying HMGB1/TLR4 Mediated Neuroinflammation in Epilepsy

HMGB1 plays a fundamental role in DNA repair, transcription, and replication. HMGB1 can be translocated to the cytosol, plasma membrane, and extracellular space in response to various stressors. When neurons, glial cells, and immune cells are stimulated by inflammatory factors (e.g., IL-1β and TNF-α) or activated in response to oxidative stress, HMGB1 is actively released from the intracellular space to the extracellular space. When an extrinsic factor (e.g., cerebral cortical dysplasia, tuberous sclerosis complex, trauma, tumor) causes cell damage or death, HMGB1 is passively released from the intracellular space to the extracellular space (16). The regulation of HMGB1 secretion is important for the regulation of HMGB1 mediated inflammation and is dependent on the corresponding factors, such as phosphorylation by calcium-dependent protein kinase C. Inflachromene is a microglial inhibitor that can bind to HMGB1, blocking its sequential cytoplasmic localization and extracellular release by perturbing the post-translational modification and downregulation of pro-inflammatory function (17). HMGB1 released extracellularly binds to TLR4 and receptors for advanced glycation end products (RAGE) on the surface of neurons and glial cells. The activated HMGB1/TLR4 signaling pathway delivers signals through protein myeloid differentiation factor 88 (MyD88) dependent and independent pathways and stimulates nuclear factors κB (NF-κB) transport into the nucleus, transcribing target genes responsible for neuroimmune inflammatory response (Figure 1) (6, 18). Upon activation by HMGB1/TLR4 signaling, phosphorylation of the NR2B subunit of the N-methyl-D-aspartic acid (NMDA) receptor leads to Ca2^+^ influx, which renders neuronal cells hyperexcitable and ultimately induces epileptogenesis (17, 19). HMGB1, binds to TLR4, a member of the danger-associated molecular pattern family (15), an endogenous danger signal (16). Pattern recognition receptors present on various immune cells can recognize danger signals such as damage-associated molecular patterns, which in turn activate the innate immune system and initiate an immunoinflammatory response (20, 21). Neuroimmune responses lead to the production and release of active inflammatory factors (such as IL-1β and HMGB1). Extracellular HMGB1 binds to TLR4 on the surface of neurons and glial cells, which in turn leads to seizure precipitation. Seizures lead to brain cell damage, which in turn promotes the passive release of HMGB1. Proinflammatory cytokines, the release of which is induced by excess HMGB1 also in turn stimulate the release of more HMGB1, playing a key role in the occurrence and perpetuation of seizures (22). Several studies have found that in kainic acid-induced animal models, an increase in extracellular HMGB1 downregulated the expression of glutamate decarboxylase 67 and glutamate dehydrogenase 1/2 and increased intracellular glutamate concentration and major histocompatibility complex II (MHC II) levels. This leads to neuronal hyperexcitability and epileptogenesis (23). Terrone et al. reported that the activation of the HMGB1/TLR4 axis modulates glutamate receptor-mediated Ca^2+^ permeability in neurons by promoting NMDA-NR2B or α-aminohydroxymethyloxazole propionic acid-GluR2 receptor phosphorylation *via* PI3-kinase (19). Another mechanism by which HMGB1 may promote epilepsy is by the destruction of the blood–brain barrier (24). HMGB1 binding to TLR4 in BBB endothelial cells and perivascular astrocytes can alter the permeability function of BBB, leading to the extravasation of serum albumin. Extravasated serum albumin activates astrocytic transforming growth factor β signaling *via* transforming growth factor β receptor type I activin receptor-like kinase 5 (Alk5), and then leads to the activation of extracellular matrix genes through the downstream MAPK pathway and Smad2/3 pathway. The activation of the extracellular matrix gene can generate transcription and translation of extracellular matrix remodeling related molecules. These cytokines are released extracellularly and promote the persistent degradation of a protective extracellular matrix structure called the periglomerular neuronal network that surrounds inhibitory interneurons. Furthermore, remodeling of these extracellular matrices leads to dysfunction of interneurons, promoting excitation/inhibition (E/I) imbalance and ultimately epileptogenesis. However, seizures per se can also cause BBB dysfunction, forming a vicious circle (25).

## Association Between HMGB1/TLR4 Mediated Neuroinflammation and Epilepsy

Several studies on acute injury-induced epilepsy animal models have shown that total HMGB1 increased in the blood before the onset of spontaneous seizures and during disease development and that both acetylated disulfide isoforms and disulfide isoforms of HMGB1 progressively increased and constituted the majority of total HMGB1 in the blood. It is speculated that HMGB1 may be involved in the initiation of epilepsy after brain injury, and the level of HMGB1 in the blood might help to identify patients with a high risk of developing spontaneous seizures early after epileptogenic injury. In addition, the release of disulfide HMGB1 in the brain following status epilepticus may contribute to epileptogenesis and the consequent onset of spontaneous seizures. However, these changes persisted only in animals with active epilepsy, and disulfide HMGB1 was not detected in the blood of healthy individuals or those with well-controlled epilepsy. Thus, HMGB1 may be a useful predictive biomarker for seizure relapse and response to therapy (26, 27).

Animals with increased HMGB1 inflammatory subtypes in the blood before the onset may prospectively identify the possibility of epilepsy later. An increase in HMGB1 inflammatory subtypes persisted during active epilepsy in these animals. An early expression of the inflammatory, pathologic disulfide isoform of HMGB1 indicates the likelihood of experiencing further seizures in patients with newly diagnosed epilepsy. The drug-refractory epilepsy patients also invariably express significantly higher acetylated disulfide isoforms which are notably absent in patients with well-controlled epilepsy. The disulfide isoform of HMGB1, which seems to be the form most likely to promote seizure generation, is a potentially novel prognostic, diagnostic, and predictive biomarker of drug-resistant epilepsy in humans and highlights potential new targets for drug development (28). Pauletti et al. found that oxidative stress occurs in the brains of patients experiencing status epilepticus, as well as in patients with drug-resistant temporal lobe epilepsy, and this phenomenon is associated with the cytoplasmic translocation of HMGB1 in neurons and glia. Therefore, inhibiting the generation of disulfide HMGB1 by reducing oxidative stress may be a potential novel therapeutic approach (26).

A clinical study suggested that increased HMGB1 or TLR4 expression correlated with a higher risk and severity of epilepsy, as well as the increased possibility of anti-seizure medication resistance (22). Additionally, activation of the HMGB1/TLR4 axis has been demonstrated in surgically resected drug-refractory brain tissue (26). Kamaşsk et al. found that HMGB-1 and TLR4 levels in the severe epilepsy group were significantly higher than those in the control group or the mild epilepsy group, and the mild epilepsy group was significantly higher than those in the control group in the serum of children aged 4–17. It has been suggested that HMGB-1 and TLR4 expression levels correlate with epilepsy severity (29). Another clinical study demonstrated that the serum concentration of HMGB1 was negatively associated with patients' intelligence scores and positively associated with the frequency of seizures and the number of epileptiform discharges. These findings suggest that serum HMGB1 is potentially involved in the initiation and progression of epilepsy or epileptic lesions and is a potential predictive factor for epilepsy prognosis (30). A study on a rat model showed that the HMGB1/TLR4 axis was overexpressed in mesial temporal lobe epilepsy and could induce neuronal synaptic reconstruction and inflammatory responses in neurons *via* the p38MAPK signaling pathway (13). The HMGB1/TLR4 pathway was upregulated in neurons and astrocytes in the brain tissues of epileptic children with FCD-II, which led to an increase in the release of downstream pro-inflammatory cytokines (31). Therefore, the HMGB1/TLR4 axis plays a key role in FCD-II-related epilepsy and mesial temporal lobe epilepsy.

In animal models of pilocarpine-induced seizure, pharmacological inactivation of HMGB1 using a monoclonal antibody reduced seizure frequency and severity, and an anti-HMGB1 monoclonal antibody exerted protective effects on neuronal apoptosis and prevented epileptogenesis in association with inhibition of HMGB1 release (27). The reported anti-epileptic effect of the anti-HMGB1 monoclonal antibody might be due to inhibition of blood–brain barrier rupture, inflammatory responses, translocation of HMGB1, and neuronal cell death. Anti-HMGB1 monoclonal antibody therapy may be a novel strategy for preventing epileptogenesis (32). Another study has incorporated both acute and chronic seizure animal models. Anti-HMGB1 monoclonal antibody treatment attenuated electroshock and pentylenetetrazol-induced acute seizures, while anti-HMGB1 monoclonal antibody did not show any anti-seizure effect in TLR4 knockout mice, supporting the fact that HMGB1-TLR4 regulatory axis contributes to epileptogenesis (33). The anti-seizure effect of the anti-HMGB1 monoclonal antibody showed sufficient potential and specificity for treating kainic acid-induced seizures in an animal model and surgical tissue slices from patients with medically refractory temporal lobe epilepsy (33). Glycyrrhizin, a glycoconjugated triterpene extracted from licorice root and a natural anti-inflammatory compound, inhibits the function of HMGB1. It has a strong neuroprotective effect in mice with experimental autoimmune encephalomyelitis by reducing HMGB1 expression and release. Thus, it can be used to treat inflammatory diseases of the central nervous system (34). Glycyrrhizin can attenuate neuronal damage and alter the disease progression of post-status epilepticus by inhibiting HMGB1 activity and its translocation and protecting the blood–brain barrier permeability in a status epilepticus model induced by lithium-pilocarpine (35). However, the potential anti-epileptic mechanism of glycyrrhizin *via* HMGB1 is still unclear in seizure models and patients. A few studies have also identified other antiepileptic treatment schemes that act on the HMGB1/TLR signaling pathway. For example, epigallocatechin gallate can significantly attenuate the increase in TLR-4 and IL-1β levels in the hippocampus to achieve anti-epileptogenesis and neuroprotective effects in pilocarpine-induced epileptic rats (36, 37). In addition, micro-RNA plays a role in HMGB1-mediated epilepsy. Both miR-129-5p and miR-542-3p inhibit the development of epilepsy by suppressing HMGB1 expression and inhibiting the TLR4/NF-kB signaling pathway (38, 39). TLR4 agonists, LPS and MPL, can attenuate seizure severity in pilocarpine-induced rats with temporal lobe epilepsy (40), and pentoxifylline may represent a promising drug to inhibit epilepsy progression by targeting the HMGB1/TLR4/RAGE signaling pathway in pentylenetetrazol (PTZ)-kindling rats (41).

## Structure and Function of IL-1β/IL-1R1

**Figure 2:**
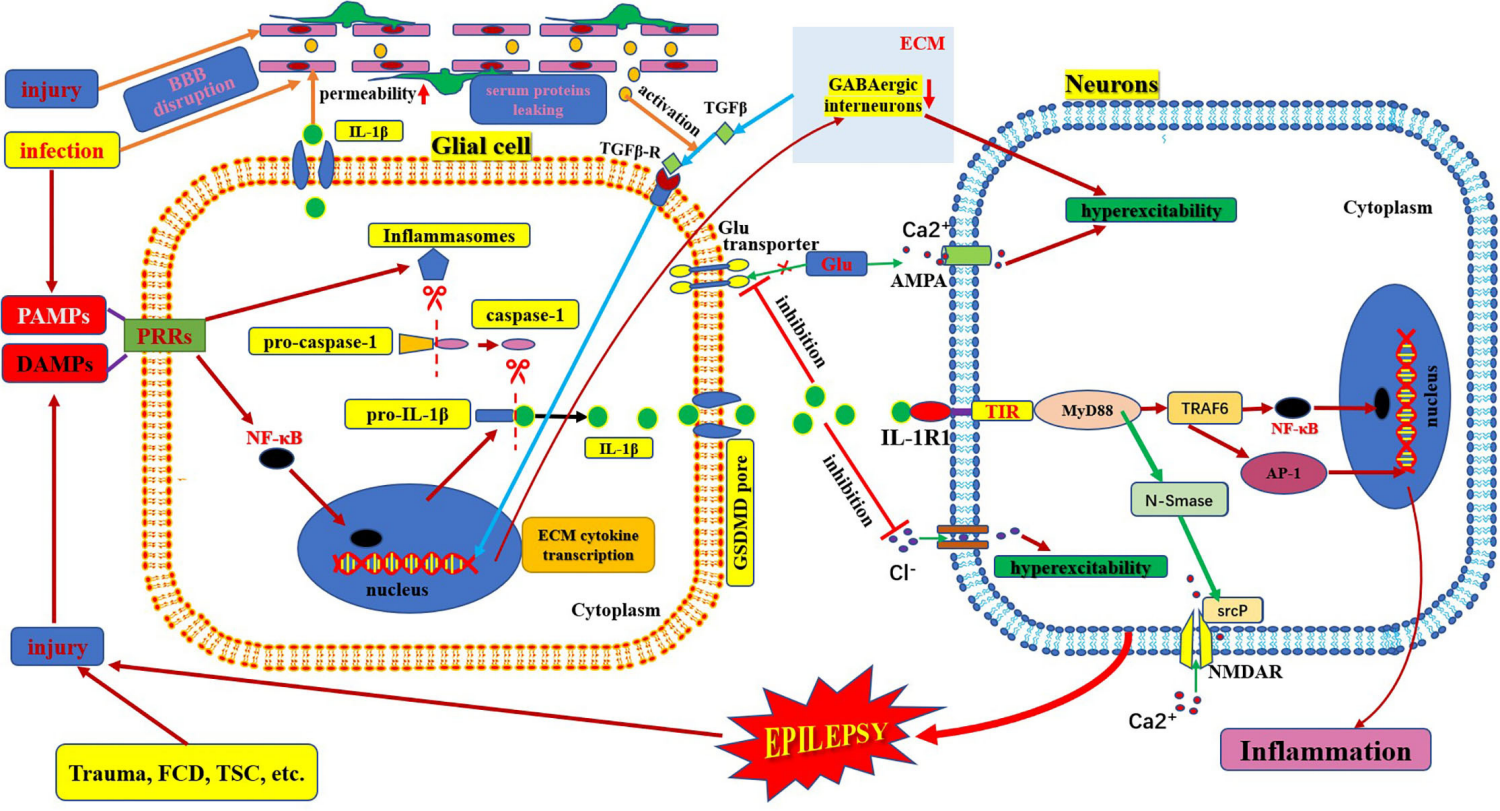
Schematic of IL-1β/IL-1R1 pathway and epilepsy. This figure shows the activation and secretion of IL-1β and its epileptogenic mechanism in combination with IL-1R1. AMPA, aminohydroxymethyloxazole propionic acid; BBB: blood–brain barrier; DAMP, danger-associated molecular patterns; FCD, focal cortical dysplasia; GLU, glutamate; IL-1β, interleukin-1β; IL-1R, interleukin-1receptor; MyD88, myeloid differentiation factor 88; NMDAR, N-methyl-D-aspartic acid receptor; NF-κB, nuclear factors κB; PAMP, pathogen-associated molecular patterns; PRR, pattern recognition receptors; TSC, tuberous sclerosis complex.

IL-1 was first identified in 1979. The IL-1 family includes three ligands, IL-1α, IL-1β, and IL-1Ra, all of which bind to the IL-1 receptor (42, 43). IL-1α is membrane-bound, whereas IL-1β is released into the extracellular space. IL1-Ra is a naturally occurring competitive inhibitor of both membrane-bound IL-1α and extracellular-released IL-1β, which acts as a brake to prevent the occurrence of excessive inflammation (44). IL1β is produced and secreted by several cell types. Although the vast majority of studies have focused on its production within cells of the innate immune system, such as monocytes and macrophages, it is mainly produced by activated microglia as well as neurons, astrocytes, and oligodendrocytes in the central nervous system (45). IL-1β is strongly induced in activated microglia and astrocytes during the acute phase of status epilepticus and in the chronic phase of spontaneous seizures in brain areas involved in seizure generation and propagation and is mainly sustained by astrocytes (4). IL-1β mainly binds to IL1R1 and activates NF-κB in target cells to induce and amplify the inflammatory response and plays an important role in injury and inflammation (Figure 2) (46).

The IL-1R family encompasses 10 type-1 transmembrane proteins with a similar architecture, which usually consist of an extracellular portion responsible for ligand binding, a transmembrane domain, and an intracellular portion where the TIR domain resides (toll IL1R homology region). IL-1R molecules can be divided into four subgroups based on their functions and differences in structural features (42). IL-1R1 is a biologically active type of IL-1R, formerly known as IL-1R type-I (47). When one of the agonist ligands, IL-1α or IL-1β, binds to IL-1R1 and changes its conformation, the complex forms of IL1-IL1R1 mediate IL-1-dependent activation (42). This complex recruits a number of intracellular adapter molecules, including myeloid differentiation factor 88, IL-1R-associated kinase, and tumor necrosis factor receptor-associated factor 6, to activate signal transduction pathways [such as nuclear factor-kB, activator protein-1, c-Jun N-terminal kinase, and p38 mitogen-associated protein kinase] and finally amplifies the inflammatory response (Figure 2) (47, 48).

## Mechanisms Underlying IL-1β/IL-1R1-Mediated Neuroimmune Inflammation in Epilepsy

IL-1β is active only after it matures and is secreted from the cell. The process of maturation and secretion of IL-1β is roughly as follows: ① the cell is infected by bacteria or cell damage occurs; ② the pathogen-associated molecular patterns or damage-associated molecular patterns bind to the pattern recognition receptors and stimulate NF-κB transport into the nucleus and induce transcription of pre-IL-1β; ③ meanwhile, after pathogen-associated molecular patterns or damage-associated molecular patterns are recognized by pattern recognition receptors, the assembly and activation of inflammasomes (cytosolic multiprotein complexes) are triggered, which can promote the transformation of pro-caspase-1 into mature caspase-1 in the cytoplasm; ④ mature active caspase-1 causes cleavage of pro-IL-1β to mature IL-β; ⑤ finally, mature IL-1β is secreted through membrane pores *via* activated gasdermin D (49–51). The mechanism of epilepsy induced by IL-1β-mediated neuroimmune inflammatory responses is complex. IL-1β levels are significantly increased in the brain tissue and plasma samples from animal models of epilepsy and patients with temporal lobe epilepsy (45, 52). The IL-1β/IL-1R1 axis enhances NMDA receptor-mediated Ca^2+^ influx into neurons *via* phosphorylation of the NR2B subunit of the NMDA receptor *via* Src kinase, resulting in excitotoxicity and seizure (19, 43). IL-1β also affects neuronal excitability by increasing the extracellular glutamate concentration by inhibiting the astroglial glutamate transporter (19). IL-1β also inhibits GABA-mediated Cl^−^ influx into neurons, leading to increased neuronal excitability (19, 44). In addition, some studies have found indirect effects on neurons through the blood-brain barrier. IL-1β/IL-1R1 is expressed during epileptogenesis in both perivascular astrocytes and endothelial cells of the blood-brain barrier (4). IL-1β can affect blood-brain barrier permeability through disruption of tight-junction organization, nitric oxide production, or matrix metalloproteinase activation in endothelial cells. The brain barrier leakage promotes the exudation of inflammatory factors and neuronal damage. Moreover, these changes could lead to the infiltration of inflammatory cells from the periphery to the brain, which can aggravate inflammation and promote hyperexcitability, excitotoxicity, and epileptogenesis (Figure 2) (43). IL-1β can also stimulate synaptophysin expression and epileptiform discharges via the PI3K/Akt/mTOR signaling pathway to induce seizure generation in an animal model with temporal lobe epilepsy (53). Furthermore, the IL-1β/IL-1R1 signaling pathway promotes glial activation, proliferation, and cytokine release, which leads to an amplified inflammatory response and increases the risk of seizures and brain damage (54). However, after epileptogenesis, microglial activation produces large amounts of inflammatory factors (such as IL-1β). The massive release of IL-1β can lead to the production of adhesion molecules by endothelial cells, increasing leukocyte infiltration, which in turn produces more inflammatory mediators, leading to neuroimmune inflammatory responses and forming a vicious cycle (45, 55).

## Association Between IL-1β/IL-1R1-Mediated Neuroimmune Inflammation and Epilepsy

Upregulation of IL-1β was found in the injured cortex, hippocampus, brain, and serum of a mouse model with post-traumatic epilepsy (54). In particular, the level of IL-1β expression in mice brains with status epilepticus was higher than that in control mice (19). Kostic *et al*. evaluated IL-1β levels in the cerebrospinal fluid and serum of 6 healthy dogs and 51 dogs with epilepsy (structural and idiopathic). IL-1β concentrations in the cerebrospinal fluid were not detectable. However, dogs with epilepsy have increased serum IL-1β levels, regardless of the underlying cause of the disease (45). IL-1β levels were increased in the brain tissue samples of patients with TLE and displayed higher plasma levels of IL-1β (52). Another clinical study demonstrated that serum concentrations of IL-1β were negatively associated with patients' intelligence scores and positively associated with the frequency of seizures and number of epileptiform discharges. These findings suggest that IL-1β is potentially involved in the initiation and progression of epilepsy or epileptic lesions and is a potential predictive factor for epilepsy prognosis (30). Kamaşsk et al. found that IL-1β levels in the severe epilepsy group were higher than those in the control group or the mild epilepsy group (*P* < 0.05), and the level in the mild epilepsy group was higher than that in the control group (*P* < 0.05); in addition, the severe epilepsy group had higher IL-1R1 than the control group in the serum of children aged 4–17. It has been suggested that IL-1β/ IL-1R1 expression correlates with epilepsy severity (29). A prospective study of epilepsy outcomes showed that serum levels of IL-1β showed a significant correlation with the measures of disease severity and the number of anti-seizure medications used and a negative correlation with the age of disease onset in children with epilepsy. Thus, these data suggest that the serum levels of IL-1β are potential prognostic biomarkers for children with epilepsy (56). Moreover, the genotype frequency of rs1143627 TT of IL-1b-31 and the homozygous IL1RN^*^I were found to be more prevalent in patients with epilepsy, and the T allele of IL-1b-31 and IL1-RAI/I was substantially positively correlated with drug resistance in those who responded well to anti-seizure medications. The genotype of IL-1β/IL-1R1 could be a predictive marker for identifying individuals at risk of seizure and drug resistance development (47).

Anakinra, a human recombinant endogenous competitive antagonist of IL-1R1, is used to treat autoinflammatory and autoimmune disorders. Repetitive intracerebroventricular injections of anakinra after electrically induced status epilepticus in rats led to a decrease in spike frequency and reduced seizure generalization during convulsive status epilepticus (19). A child with febrile infection-related epilepsy syndrome (FIRES) showed improvement with anakinra and super-refractory status epilepticus (57). In addition, an adolescent female with a diagnosis of refractory epilepsy was treated with anakinra first and then with canakinumab, an IL-1β antibody, during a nonconvulsive status epilepticus, which resulted in complete resolution of clinical seizures (58). The IL-1 β/IL-1R signaling pathway might be a profoundly impactful adjunctive medication for certain refractory epilepsy syndromes.

## Association of HMGB1/TLR4 and IL-1β/IL-1R

**Figure 3:**
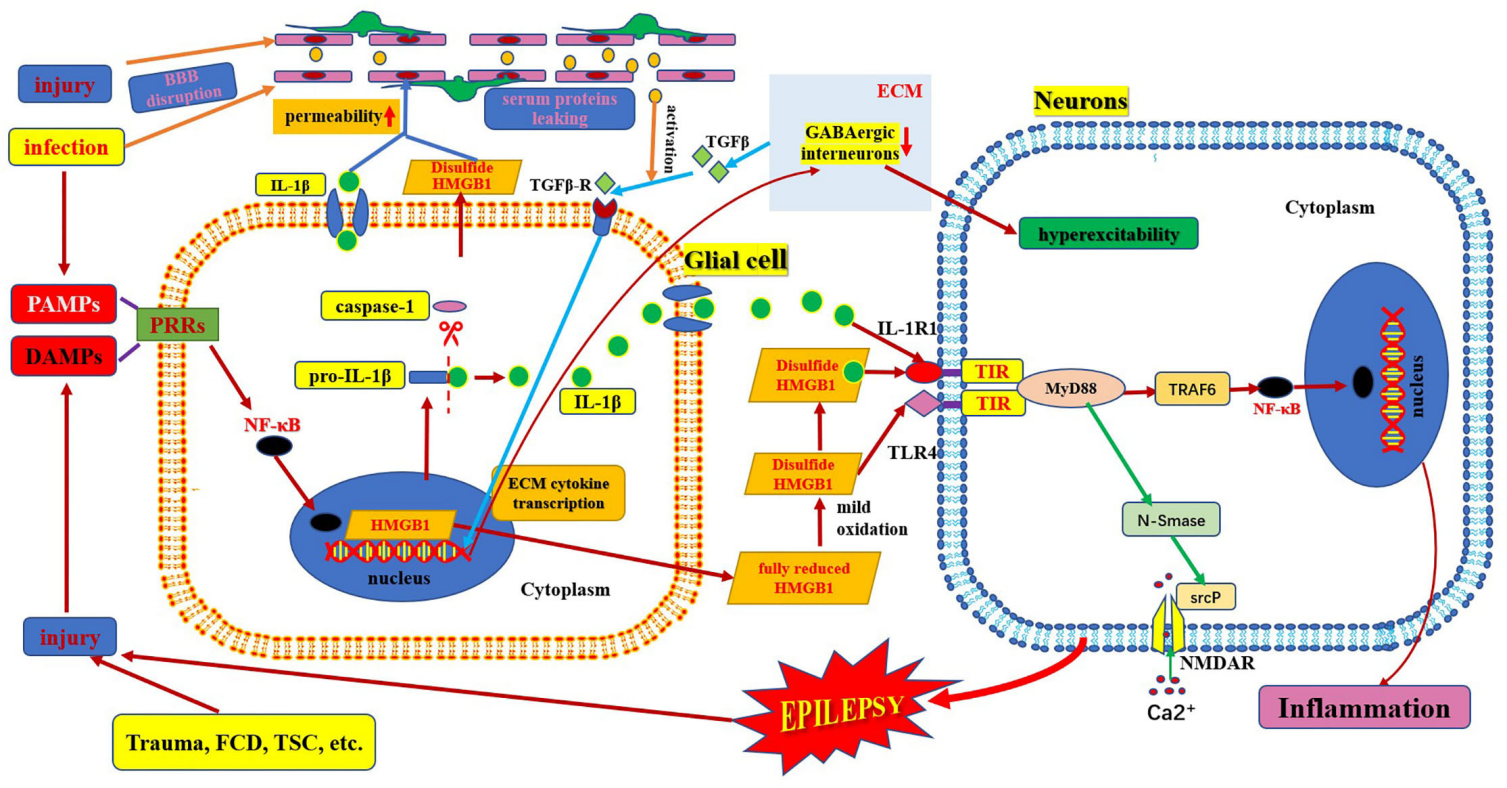
Schematic of interaction of HMGB1/TLR-4 and IL-1β/IL-1R pathway in epilepsy. This figure shows the interaction between HMGB1/TLR-4 and the IL-1β/IL-1R pathway during their activation, secretion, and receptor combinations, and in the epileptogenic mechanism. BBB, blood–brain barrier; DAMP, danger-associated molecular patterns; FCD, focal cortical dysplasia; IL-1β, interleukin-1β; IL-1R, interleukin-1receptor; HMGB1, high mobility group box1; IL-1β, interleukin-1β; MyD88, myeloid differentiation factor 88; NMDAR, N-methyl-D-aspartic acid receptor; NF-κB, nuclear factors κB; PAMP, pathogen-associated molecular patterns; PRR, pattern recognition receptors; TLR, toll-like receptor; TSC, tuberous sclerosis complex.

The HMGB1/TLR4 and IL-1β/IL-1R1 signaling pathways are key upstream generators of the neuroinflammatory response. HMGB1 can also bind to IL-1β to initiate an IL-1R1-mediated proinflammatory response (6, 17). There is a common crucial signaling domain in both the IL-1 receptor and TLR4, designated as the toll interleukin-1 receptor homology region, which is now known as the TIR domain (42). Thus, the downstream signaling pathways activated by the TIR domain-containing receptor activation are very similar. First, several MyD88 molecules are recruited to the TIR domain to form MyD88 oligomers and then, IRAK-4, IRAK-1, and/or IRAK-2 are recruited. TRAF6 is recruited to hyperphosphorylated IRAK-1 oligomers and is activated. TAB2 and TAB3, ubiquitinated by TRAF6, enter the complex and associate with the TAK1/TAB1 complex, and undergo a conformational change resulting in TAK1 auto-phosphorylation and activation (Figure 3). Active TAK1 phosphorylates and activates the downstream protein kinase IKK, and NF-κB is then transported to the nucleus (14, 42). Eventually, their activation excited endogenous ligands leading to the transcriptional induction of NF-kB-regulated inflammatory genes, and, as a consequence, generating and rapidly amplifying the inflammatory cascade (46, 59). Activation of HMGB1/TLR4 and IL-1β/IL-1R1 axis-in depolarized neurons promotes excitotoxicity and seizures by enhancing Ca^2+^ influx *via* NMDA receptors (8). Another study showed that the activation of IL-1R1 and TLR4 signaling may induce acquired channelopathies by reducing the cyclic AMP-gated channel type 1 protein level and channel-mediated conductance on dendrites of hippocampal pyramidal neurons (60). Cyclic AMP-gated channel type 1 is a key regulator of the filtering properties of hippocampal pyramidal cell dendrites and their responses to excitatory inputs. They are involved in theta rhythms, which in turn are linked to cognitive functions. Cyclic AMP-gated channel type 1 is downregulated in animal models and human epilepsy and contributes to seizures and cognitive deficits (19). Moreover, both HMGB1/TLR4 and IL-1β/IL-1R signaling pathways are involved and contribute to the increased permeability of the blood–brain barrier (Figure 3). Leaky serum proteins induce transcription and translation of extracellular matrix-associated cytokines *via* transforming growth factors in astrocytes β signaling. These cytokines remodel the extracellular matrix. The ongoing degradation of perineuronal nets (a protective extracellular matrix) around GABAergic interneurons causes GABAergic interneuron dysfunction, which may contribute to hyperexcitability of brain tissue and provoke a long-lasting decrease in seizure threshold (25, 46).

## Future Perspective and Conclusion

Preclinical studies and clinical evidence show that HMGB1/TLR4 and IL-1β/IL-1R1 signaling pathways are involved in epileptogenesis caused by neuroimmune inflammatory responses and participate in the neuroinflammatory response of brain injury after epilepsy (Table 1). They are closely related to the mechanism of epilepsy and the development of drug resistance and are important biomarkers for the occurrence, prognosis, and prediction of epilepsy. These two signaling pathways can induce neuroimmune inflammatory responses through multiple pathways, which is an important part of the neuroimmune inflammatory response theory in the pathogenesis of epilepsy. Notably, research on antibodies or inhibitors against these two signaling pathways has also made some progress and has shown good therapeutic effects. However, the complete upstream and downstream links of HMGB1/TLR4 and IL-1β/IL-1R1 signaling pathways, their complete mechanism of action in epilepsy of different etiologies, and the participation of genetic factors are still very limited. At present, there are few studies on the relationship between these two signaling pathways and their related antibodies or inhibitors, and there is a lack of in-depth experiments based on different etiologies and animal models. The clinical applications of these drugs require further exploration. Finally, it is expected that the IL-1β/Il-1R1 and HMGB1/TLR4 signaling pathways will provide more in-depth, scientific, and comprehensive research on the occurrence, prediction, prognosis, and treatment of epilepsy.

**Table 1:** Summary of studies on the HMGB1/TLR4 and IL-1β/ IL-1R pathways and their association with epilepsy.

**S.N**.	**Intervention**	**Experimental models**	**Pathway**	**Observations**	**References**
1	HMGB1/TLR4	① Surgically removed brain sample of patients with intractable epilepsy ② Cell model of epilepsy induced by coriaria lactone (CL)	TLR4/NF-κB	① Increased expression of HMGB1 and NF-κB ② Translocation of HMGB1 from nuclear to cytoplasmic	(6)
2	HMGB1/TLR4	① Surgically removed brain tissue of children with drug-resistant MTLE ② Pilocarpine induced SE in rats	p38MAPK	① Overexpression of HMGB1 and TLR4 ② HMGB1 upregulated the protein level of p38MAPK	(13)
3	HMGB1/TLR4	Peripheral venous blood samples of patients with epilepsy	-	① Expressions of HMGB1 and TLR4 were higher in epilepsy patients ② Elevated HMGB1 and TLR4 expressions were both associated with longer seizure duration and increased seizure frequency ③ increased HMGB1 and TLR4 expressions were correlated with a higher possibility of anti-epilepsy drugs resistance	(22)
4	HMGB1	Primary rat neural cells (PRNCs) by KA administration	GAD67 and GLUD 1/2	① KA induced the translocation of HMGB1 from the nucleus to the cytosol ② PRNC cell viability and mitochondrial Activity ↓ ③ Expression of GAD67s and GLUD1/2 ↓	(23)
5	Anti-HMGB1 monoclonal antibody	① Acute seizure model induced by maximal electroshock or PTZ in rats ② chronic seizure model induced by KA in rats ③ Surgical specimens of patients with intractable epilepsy	-	① Acute seizures and translocation of HMGB1 ↓ ② The severity of chronic epilepsy ↓ ③ Spontaneous discharges ↓	(33)
6	Glycyrrhizin(GL)	Pilocarpine induced SE in rats	-	① HMGB1 expression in serum and hippocampus in the GL treatment group ↓ ② HMGB1 translocation from the nucleus to cytoplasm in hippocampal in the GL treatment group ↓ ③ Neuronal damage in the hippocampus in the GL treatment group ↓	(35)
7	Epigallocatechin-3-allate(EGCG)	Pilocarpine induced SE in rats	TLR4/NF-κB	① The frequency of spontaneous recurrent seizures and duration of seizures ↓ ② Level of TLR4 and NF-κB in the EGCG treatment group ↓	(37)
8	Lipopolysaccharides and Monophosphoryl lipid A	Pilocarpine induced seizure in rats	-	Early preconditioning with TLR4 agonists attenuates seizure severity	(40)
9	Pentoxifylline(PTX)	Pentylenetetrazole (PTZ)-induced seizure in rats	HMGB1/ RAGE/ TLR4	① Seizure severity score in the PTX treatment group ↓ ② Level of HMGB1, TLR4, RAGE, and NF-κB in the PTX treatment group ↓ ③ Cognition improved in the PTX treatment group ↑	(41)
10	IL-1β	Dogs with epilepsy (structural and idiopathic)	-	① Serum IL-1β was not elevated in dogs with TBI ② Increased serum IL-1β in dogs with epilepsy	(45)
11	IL-1β, caspase-1	① Hippocampal tissues from patients with MTLE ② Plasma of patients with MTLE	-	Both of them increased in tissue samples and plasma of patients with TLE	(52)
12	IL-1β	① Pilocarpine induced SE in rats ② Hippocampal neuronal model	PI3K/Akt/mTOR	IL-1β promoted SYN expression, and SYN expression is related to the PI3K/Akt/mTOR pathway	(53)
13	IL-1 receptor antagonist (IL-1Ra)	Pediatric mouse model with epilepsy after traumatic brain injury	-	① rIL-1Ra reduces subacute seizure susceptibility after pTBI ② rIL-1Ra reduces the chronic PTZ-evoked seizure response	(54)
14	IL-1β	Peripheral venous blood samples of children with epilepsy	-	① Age at disease onset showed a significant correlation negatively with serum levels of IL-1β ② Serum levels of IL-1β showed a positive correlation with the measures of disease severity	(56)
15	Anakinra	A 13-year-old child with febrile infection-related epilepsy syndrome (FIRES)	-	Aanakinra reduces the relapse of highly recurrent refractory seizures at 1.5 years after FIRES onset	(57)
16	Anakinra canakinumab	A 14-year-old female with systemic autoinflammation with intractable epilepsy	-	Near-complete resolution of clinical seizures	(58)
17	Fisetin	① Acute seizure model induced by maximal electroshock or PTZ in rats ② chronic seizure model induced by PTZ in subconvulsive dose in rats	-	① delayed onset of seizures ② decreased the percentage of fully kindled mice ③ attenuated seizure severity score and mortality ④ decreased levels of HMGB1, TLR-4, IL-1β, and IL-1R1 in the hippocampus and cortex of the kindled mice	(59)

## Author Contributions

SL: conceptualization, writing—reviewing and editing, and funding acquisition. SZ and FC: writing—original draft preparation and investigation. FZ: writing—reviewing and editing. All authors contributed to the article and approved the submitted version.

## Funding

This research was supported by the Beijing Nature & Science Foundation of China (7202045, SL) and the National Nature and Science Foundation of China (82071448, SL). The funders were not involved in the study design, data collection and analysis, interpretation of data, or writing of the report.

## Conflict of Interest

The authors declare that the research was conducted in the absence of any commercial or financial relationships that could be construed as a potential conflict of interest.

## Publisher's Note

All claims expressed in this article are solely those of the authors and do not necessarily represent those of their affiliated organizations, or those of the publisher, the editors and the reviewers. Any product that may be evaluated in this article, or claim that may be made by its manufacturer, is not guaranteed or endorsed by the publisher.

## References

[1] Trinka E Kwan P Lee B Dash A. Epilepsy in Asia: Disease burden, management barriers, and challenges. Epilepssia. (2019) 60(Suppl 1):7–21. 10.1111/epi.14458 29953579

[2] Thijs RD Surges R O'Brien TJ Sander JW. Epilepsy in adults. Lancet. (2019) 393:689–701. 10.1016/S0140-6736(18)32596-0 30686584

[3] Wang S Zhao M Li T Zhang C Zhou J Wang M. Long-term efficacy and cognitive effects of bilateral hippocampal deep brain stimulation in patients with drug-resistant temporal lobe epilepsy. Neurol Sci. (2021) 42:225–33. 10.1007/s10072-020-04554-8 32632633

[4] van Vliet EA Aronica E Vezzani A Ravizza T. Review: Neuroinflammatory pathways as treatment targets and biomarker candidates in epilepsy: emerging evidence from preclinical and clinical studies. Neuropathol Appl Neurobiol. (2018) 44:91–111. 10.1111/nan.12444 28977690

[5] Korff CM Dale RC. The immune system in pediatric seizures and epilepsies. Pediatrics. (2017) 140:e20163534. 10.1542/peds.2016-3534 28794080

[6] Shi Y Zhang L Teng J Miao W. HMGB1 mediates microglia activation via the TLR4/NF-κB pathway in coriaria lactone induced epilepsy. Mol Med Rep. (2018) 17:5125–31. 10.3892/mmr.2018.8485 29393419 PMC5865977

[7] Yang L Wang F Yang L Yuan Y Chen Y Zhang G. HMGB1 a-box reverses brain edema and deterioration of neurological function in a traumatic brain injury mouse model cell. Physiol Biochem. (2018) 46:2532–42. 10.1159/000489659 29742510

[8] Paudel YN Semple BD Jones NC Othman I Shaikh MF. High mobility group box 1 (HMGB1) as a novel frontier in epileptogenesis: from pathogenesis to therapeutic approaches. J Neurochem. (2019) 151:542–57. 10.1111/jnc.14663 30644560

[9] Andersson U Tracey KJ Yang H. Post-translational modification of HMGB1 disulfide bonds in stimulating and inhibiting inflammation. Cells. (2021) 10:3323. 10.3390/cells10123323 34943830 PMC8699546

[10] Zhao L Liu P Kepp O Kroemer G. Methods for measuring HMGB1 release during immunogenic cell death. Methods Enzymol. (2019) 629:177–93. 10.1016/bs.mie.2019.05.001 31727240

[11] Hubert P Roncarati P Demoulin S Pilard C Ancion M Reynders C. Extracellular HMGB1 blockade inhibits tumor growth through profoundly remodeling immune microenvironment and enhances checkpoint inhibitor-based immunotherapy. J Immunother Cancer. (2021) 9:e001966. 10.1136/jitc-2020-001966 33712445 PMC7959241

[12] Vijay K. Toll-like receptors in immunity and inflammatory diseases: past, present, and future. Int Immunopharmacol. (2018) 59:391–412. 10.1016/j.intimp.2018.03.002 29730580 PMC7106078

[13] Yang W Li J Shang Y Zhao L Wang M Shi J. HMGB1-TLR4 axis plays a regulatory role in the pathogenesis of mesial temporal lobe epilepsy in immature rat model and children via the p38MAPK signaling pathway. Neurochem Res. (2017) 42:1179–90. 10.1007/s11064-016-2153-0 28176142

[14] Fitzgerald KA Kagan JC. Toll-like receptors and the control of immunity. Cell. (2020) 180:1044–66. 10.1016/j.cell.2020.02.041 32164908 PMC9358771

[15] Paudel YN Angelopoulou E Akyuz E Piperi C Othman I Shaikh MF. Role of Innate Immune Receptor TLR4 and its endogenous ligands in epileptogenesis. Pharmacol Res. (2020) 160:105172. 10.1016/j.phrs.2020.105172 32871246

[16] Wen Q Liu J Kang R Zhou B Tang D. The release and activity of HMGB1 in ferroptosis. Biochem Biophys Res Commun. (2019) 510:278–83. 10.1016/j.bbrc.2019.01.090 30686534

[17] Abg Abd Wahab DY Gau CH Zakaria R Muthu Karuppan MK A-Rahbi BS Abdullah Z. Review on cross talk between neurotransmitters and neuroinflammation in striatum and cerebellum in the mediation of motor behaviour. Biomed Res Int. (2019) 2019:1767203. 10.1155/2019/1767203 31815123 PMC6877979

[18] Paudel YN Shaikh MF Chakraborti A Kumari Y Aledo-Serrano Á Aleksovska K. HMGB1: a common biomarker and potential target for tbi, neuroinflammation, epilepsy, and cognitive dysfunction. Front Neurosci. (2018) 12:628. 10.3389/fnins.2018.00628 30271319 PMC6142787

[19] Terrone G Balosso S Pauletti A Ravizza T Vezzani A. Inflammation and reactive oxygen species as disease modifiers in epilepsy. Neuropharmacology. (2020) 167:107742. 10.1016/j.neuropharm.2019.107742 31421074

[20] Amarante-Mendes GP Adjemian S Branco LM Zanetti LC Weinlich R Bortoluci KR. Pattern recognition receptors and the host cell death molecular machinery. Front Immunol. (2018) 9:2379. 10.3389/fimmu.2018.02379 30459758 PMC6232773

[21] Mohseni-Moghaddam P Roghani M Khaleghzadeh-Ahangar H Sadr SS Sala C. A literature overview on epilepsy and inflammasome activation. Brain Res Bull. (2021) 172:229–35. 10.1016/j.brainresbull.2021.05.001 33964347

[22] Kan M Song L Zhang X Zhang J Fang P. Circulating high mobility group box-1 and toll-like receptor 4 expressions increase the risk and severity of epilepsy. Braz J Med Biol Res. (2019) 52:e7374. 10.1590/1414-431X20197374 31241711 PMC6596364

[23] Kaneko Y Pappas C Malapira T Vale FL Tajiri N Borlongan CV. Extracellular HMGB1 modulates glutamate metabolism associated with kainic acid-induced epilepsy-like hyperactivity in primary rat neural cells. Cell Physiol Biochem. (2017) 41:947–59. 10.1159/000460513 28222432

[24] Liebner S Dijkhuizen RM Reiss Y Plate KH Agalliu D Constantin G. Functional morphology of the blood-brain barrier in health and disease. Acta Neuropathol. (2018) 135:311–36. 10.1007/s00401-018-1815-1 29411111 PMC6781630

[25] Kim SY Senatorov VV Jr Morrissey CS Lippmann K Vazquez O Milikovsky DZ. TGFβ signaling is associated with changes in inflammatory gene expression and perineuronal net degradation around inhibitory neurons following various neurological insults. Sci Rep. (2017) 7:7711. 10.1038/s41598-017-07394-3 28794441 PMC5550510

[26] Pauletti A Terrone G Shekh-Ahmad T Salamone A Ravizza T Rizzi M. Targeting oxidative stress improves disease outcomes in a rat model of acquired epilepsy. Brain. (2019) 142:e39. 10.1093/brain/awz130 31145451 PMC6598637

[27] Walker LE Frigerio F Ravizza T Ricci E Tse K Jenkins RE. Molecular isoforms of high-mobility group box 1 are mechanistic biomarkers for epilepsy. J Clin Invest. (2019) 129:2166. 10.1172/JCI129285 30958803 PMC6486347

[28] Auvin S Walker L Gallentine W Jozwiak S Tombini M Sills GJ. Prospective clinical trials to investigate clinical and molecular biomarkers. Epilepsia. (2017) 58 Suppl 3:20–6. 10.1111/epi.13782 28675556

[29] Kamaşak T Dilber B Yaman SÖ Durgut BD Kurt T Çoban E. HMGB-1, TLR4, IL-1R1, TNF-α, and IL-1β: novel epilepsy markers? Epileptic Disord. (2020) 22:183–93. 10.1684/epd.2020.1155 32301731

[30] Zhu M Chen J Guo H Ding L Zhang Y Xu Y. high mobility group protein B1 (HMGB1) and Interleukin-1β as prognostic biomarkers of epilepsy in children. J Child Neurol. (2018) 33:909–17. 10.1177/0883073818801654 30303442

[31] Zhang Z Liu Q Liu M Wang H Dong Y Ji T. Upregulation of HMGB1-TLR4 inflammatory pathway in focal cortical dysplasia type II. J Neuroinflammation. (2018) 15:27. 10.1186/s12974-018-1078-8 29382328 PMC5791174

[32] Ravizza T Terrone G Salamone A Frigerio F Balosso S Antoine DJ. High Mobility Group Box 1 is a novel pathogenic factor and a mechanistic biomarker for epilepsy. Brain Behav Immun. (2018) 72:14–21. 10.1016/j.bbi.2017.10.008 29031614

[33] Zhao J Wang Y Xu C Liu K Wang Y Chen L. Therapeutic potential of an anti-high mobility group box-1 monoclonal antibody in epilepsy. Brain Behav Immun. (2017) 64:308–19. 10.1016/j.bbi.2017.02.002 28167116

[34] Sun Y Chen H Dai J Wan Z Xiong P Xu Y. Glycyrrhizin protects mice against experimental autoimmune encephalomyelitis by inhibiting high-mobility group Box 1 (HMGB1) expression and neuronal HMGB1 release. Front Immunol. (2018) 9:1518. 10.3389/fimmu.2018.01518 30013568 PMC6036111

[35] Li YJ Wang L Zhang B Gao F Yang CM. Glycyrrhizin, an HMGB1 inhibitor, exhibits neuroprotective effects in rats after lithium-pilocarpine-induced status epilepticus. J Pharm Pharmacol. (2019) 71:390–99. 10.1111/jphp.13040 30417405

[36] Khalatbary AR Khademi E. The green tea polyphenolic catechin epigallocatechin gallate and neuroprotection. Nutr Neurosci. (2020) 23:281–94. 10.1080/1028415X.2018.1500124 30043683

[37] Qu Z Jia L Xie T Zhen J Si P Cui Z. (-)-Epigallocatechin-3-gallate protects against lithium-pilocarpine-induced epilepsy by inhibiting the toll-like receptor 4 (TLR4)/nuclear factor-κB (NF-κB) signaling pathway. Med Sci Monit. (2019) 25:1749–58. 10.12659/MSM.915025 30843525 PMC6417148

[38] Liu AH Wu YT Wang YP. MicroRNA-129-5p inhibits the development of autoimmune encephalomyelitis-related epilepsy by targeting HMGB1 through the TLR4/NF-kB signaling pathway. Brain Res Bull. (2017) 132:39–149. 10.1016/j.brainresbull.2017.05.004 28528202

[39] Yan Y Xia H Hu J Zhang B. MicroRNA-542-3p Regulates P-glycoprotein expression in rat epilepsy via the toll-like receptor 4/nuclear Factor-kappaB signaling pathway. Curr Neurovasc Res. (2019) 16:433–40. 10.2174/1567202616666191023160201 31702493

[40] Hosseinzadeh M Pourbadie HG Khodagholi F Daftari M Naderi N Motamedi F. Preconditioning with toll-like receptor agonists attenuates seizure activity and neuronal hyperexcitability in the pilocarpine rat model of epilepsy. Neuroscience. (2019) 408:388–99. 10.1016/j.neuroscience.2019.04.020 31026566

[41] Badawi GA Shokr MM Zaki HF Mohamed AF. Pentoxifylline prevents epileptic seizure via modulating HMGB1/RAGE/TLR4 signalling pathway and improves memory in pentylenetetrazol kindling rats. Clin Exp Pharmacol Physiol. (2021) 48:1111–24. 10.1111/1440-1681.13508 33899956

[42] Boraschi D Italiani P Weil S Martin MU. The family of the interleukin-1 receptors. Immunol Rev. (2018) 281:197–232. 10.1111/imr.12606 29248002

[43] Zhand A Sayad A Ghafouri-Fard S Arsang-Jang S Mazdeh M Taheri M. Expression analysis of GRIN2B, BDNF, and IL-1β genes in the whole blood of epileptic patients. Neurol Sci. (2018) 39:1945–53. 10.1007/s10072-018-3533-9 30140987

[44] Barseem NF Khattab ESAEH Mahasab MM. IL-1β-31/IL1-RA genetic markers association with idiopathic generalized epilepsy and treatment response in a cohort of Egyptian population. Int J Neurosci. (2020) 130:348–54. 10.1080/00207454.2019.1688809 31698971

[45] Kostic D Carlson R Henke D Rohn K Tipold A. Evaluation of IL-1β levels in epilepsy and traumatic brain injury in dogs. BMC Neurosci. (2019) 20:29. 10.1186/s12868-019-0509-5 31208341 PMC6580646

[46] Webster KM Sun M Crack P O'Brien TJ Shultz SR Semple BD. Inflammation in epileptogenesis after traumatic brain injury. J Neuroinflammation. (2017) 14:10. 10.1186/s12974-016-0786-1 28086980 PMC5237206

[47] Arend WP Palmer G Gabay C. IL-1, IL-18, and IL-33 families of cytokines. Immunol Rev. (2008) 223:20–38. 10.1111/j.1600-065X.2008.00624.x 18613828

[48] Chen L Zheng L Chen P Liang G. Myeloid differentiation primary response protein 88 (MyD88): the central hub of TLR/IL-1R signaling. J Med Chem. (2020) 63:13316–29. 10.1021/acs.jmedchem.0c00884 32931267

[49] Voet S Srinivasan S Lamkanfi M van Loo G. Inflammasomes in neuroinflammatory and neurodegenerative diseases. EMBO Mol Med. (2019) 11:e10248. 10.15252/emmm.201810248 31015277 PMC6554670

[50] Zhou Z Li H Tian S Yi W Zhou Y Yang H. Critical roles of NLRP3 inflammasome in IL-1β secretion induced by Corynebacterium pseudotuberculosis in vitro. Mol Immunol. (2019) 116:11–7. 10.1016/j.molimm.2019.09.016 31563023

[51] Lopez-Castejon G Brough D. Understanding the mechanism of IL-1β secretion. Cytokine Growth Factor Rev. (2011) 22:189–95. 10.1016/j.cytogfr.2011.10.001 22019906 PMC3714593

[52] Cristina de Brito Toscano E Leandro Marciano Vieira É Boni Rocha Dias B Vidigal Caliari M Paula Gonçalves A Varela Giannetti A. NLRP3 and NLRP1 inflammasomes are up-regulated in patients with mesial temporal lobe epilepsy and may contribute to overexpression of caspase-1 and IL-β in sclerotic hippocampi. Brain Res. (2021) 1752:147230. 10.1016/j.brainres.2020.147230 33385378

[53] Xiao Z Peng J Wu L Arafat A Yin F. The effect of IL-1β on synaptophysin expression and electrophysiology of hippocampal neurons through the PI3K/Akt/mTOR signaling pathway in a rat model of mesial temporal lobe epilepsy. Neurol Res. (2017) 39:640–48. 10.1080/01616412.2017.1312070 28372486

[54] Semple BD O'Brien TJ Gimlin K Wright DK Kim SE Casillas-Espinosa PM. Interleukin-1 receptor in seizure susceptibility after traumatic injury to the pediatric brain. J Neurosci. (2017) 37:7864–77. 10.1523/JNEUROSCI.0982-17.2017 28724747 PMC5559762

[55] Lan X Han X Li Q Yang QW Wang J. Modulators of microglial activation and polarization after intracerebral haemorrhage. Nat Rev Neurol. (2017) 13:420–33. 10.1038/nrneurol.2017.69 28524175 PMC5575938

[56] Choi J Kim SY Kim H Lim BC Hwang H Chae JH. Serum α-synuclein and IL-1β are increased and correlated with measures of disease severity in children with epilepsy: potential prognostic biomarkers? BMC Neurol. (2020) 20:85. 10.1186/s12883-020-01662-y 32151248 PMC7061464

[57] Dilena R Mauri E Aronica E Bernasconi P Bana C Cappelletti C. Therapeutic effect of Anakinra in the relapsing chronic phase of febrile infection-related epilepsy syndrome. Epilepsia Open. (2019) 4:344–50. 10.1002/epi4.12317 31168503 PMC6546072

[58] DeSena AD Do T Schulert GS. Systemic autoinflammation with intractable epilepsy managed with interleukin-1 blockade. J Neuroinflammation. (2018) 15:38. 10.1186/s12974-018-1063-2 29426321 PMC5807745

[59] Khatoon S Agarwal NB Samim M Alam O. Neuroprotective effect of fisetin through suppression of IL-1R/TLR axis and apoptosis in pentylenetetrazole-induced kindling in mice. Front Neurol. (2021) 12:689069. 10.3389/fneur.2021.689069 34354662 PMC8333701

[60] Frigerio F Flynn C Han Y Lyman K Lugo JN Ravizza T. Neuroinflammation alters integrative properties of rat hippocampal pyramidal cells. Mol Neurobiol. (2018) 55:7500–11. 10.1007/s12035-018-0915-1 29427087 PMC6070409

